# A Multi-Scale Recursive Attention Feature Fusion Network for Image Super-Resolution Reconstruction Algorithm

**DOI:** 10.3390/s23239458

**Published:** 2023-11-28

**Authors:** Xiaowei Han, Lei Wang, Xiaopeng Wang, Pengchao Zhang, Haoran Xu

**Affiliations:** The Key Laboratory of Industrial Automation of Shaanxi Province, Shaanxi University of Technology, Hanzhong 723000, China; hxw_1405@163.com (X.H.); 18235277605@163.com (X.W.); snutzpc@126.com (P.Z.); 19862168210@163.com (H.X.)

**Keywords:** super-resolution reconstruction, multi-scale feature, recursive networks, attention feature fusion

## Abstract

In recent years, deep convolutional neural networks (CNNs) have made significant progress in single-image super-resolution (SISR) tasks. Despite their good performance, the single-image super-resolution task remains a challenging one due to problems with underutilization of feature information and loss of feature details. In this paper, a multi-scale recursive attention feature fusion network (MSRAFFN) is proposed for this purpose. The network consists of three parts: a shallow feature extraction module, a multi-scale recursive attention feature fusion module, and a reconstruction module. The shallow features of the image are first extracted by the shallow feature extraction module. Then, the feature information at different scales is extracted by the multi-scale recursive attention feature fusion network block (MSRAFFB) to enhance the channel features of the network through the attention mechanism and fully fuse the feature information at different scales in order to improve the network’s performance. In addition, the image features at different levels are integrated through cross-layer connections using residual connections. Finally, in the reconstruction module, the upsampling capability of the deconvolution module is used to enlarge the image while extracting its high-frequency information in order to obtain a sharper high-resolution image and achieve a better visual effect. Through extensive experiments on a benchmark dataset, the proposed network model is shown to have better performance than other models in terms of both subjective visual effects and objective evaluation metrics.

## 1. Introduction

Image super-resolution reconstruction (SR) is an important image processing technique that has far-reaching significance in the field of computer vision. Super-resolution techniques enhance low-resolution (LR) images by transforming them into high-resolution (HR) counterparts [1,2,3], thereby improving the quality of the images. Image super-resolution techniques are widely used in medical imaging [4,5], video security imaging [6], satellite remote sensing [7,8], image spectroscopy [9,10], and other fields with important applications. However, image super-resolution reconstruction is an ill-posed problem, as a low-resolution image can be obtained from multiple HR images by various methods; solving this problem requires high performance on the part of the algorithm [11]. To solve this problem, researchers have proposed various super-resolution reconstruction methods. There are currently three main types of super-resolution reconstruction methods: interpolation-based methods [12], reconstruction-based methods [13], and deep learning-based methods [14,15]. Interpolation-based approaches upsample the LR image through a simple interpolation technique; however, they cannot obtain the real-time detail information of the image. Reconstruction-based methods reconstruct LR images by means of image modeling or optimization problems, however, they consume a large amount of computing power. The learning-based approach uses deep learning networks to learn and predict HR images from a large number of HR/LR image pairs with good reconstruction and fast speed, and is currently the research direction with the most potential.

Recently, deep learning-based super-resolution reconstruction algorithms have become a hot research topic in academia. Dong et al. [16] first proposed a CNN-based SISR method called SRCNN, which is an end-to-end three-layer neural network. However, this simple network structure does not handle information-rich images well. Therefore, many researchers have started to propose deeper and wider neural networks to improve network performance [17,18,19,20,21,22,23,24,25,26,27,28,29]. For example, RCAN [17] is a deep network with a depth of more than 400 layers. Although it shows superior performance, the significant increase in network depth gives rise to problems such as increased network complexity and computation cost, which increase the performance requirements of hardware devices.

Researchers have been able to reduce the network parameters while deepening the network structure to achieve better performance. For example, IDN [18] has been proposed as a fast and accurate SISR network with information distillation. DSRN [19] uses a dual-state recurrent network, which has advantages in terms of performance. Recursive networks are an effective network shrinkage model that can effectively extract and fuse features in images to obtain more accurate and comprehensive feature representation while improving the image reconstruction capability of the network. However, the above-mentioned SISR methods seem to lack flexibility in dealing with spatial contexts of different scales, and cannot effectively extract and use local and global features, making it necessary to capture both local and global contextual information to make full use of the extracted feature information.

In order to deal with these problems, we propose a multi-scale recursive attention feature fusion network (MSRAFFN) with the aim of improving the performance of image super-resolution reconstruction through effective use of multi-scale features. The proposed network uses a multi-scale recursive attention feature fusion block (MSRAFFB) consisting of a multi-scale feature extraction unit (MSFE) and an attention feature fusion unit (AFF), with the MSFE unit consisting of multiple branches for extracting features at different scales. The AFF unit is used to adaptively fuse multi-scale features. Meanwhile, residual connections are used in the backbone network to integrate the layered features from the multi-scale feature extraction module with the deeper features through cross-layer connections, thereby preserving more detailed information of the image. In summary, MSRAFFN can better recover details in HR images by extracting more contextual information from LR images, resulting in improved image reconstruction quality. The contributions of this paper are as follows:

(1) An MSRAFFN is proposed that utilizes a multi-scale recursive network structure and attention mechanism to govern network information exchange and effectively handle feature information at different scales.
(2) An MSFE block is proposed to extract feature information from different levels in the form of parallel branch stacked connections, then fuse features between each level layer-by-layer to obtain a reconstructed image with richer texture information.
(3) The AFF block is designed to integrate feature information from each branch within the MSRAFFB and learn the importance of the weights of the features of different branches using the attention mechanism.
(4) The proposed network directly extracts feature information from the LR image without interpolation, uses local residuals to connect neighboring layers within the MSRAFFB to facilitate information transfer within a single block, and utilizes recursion and global residuals to facilitate information exchange between blocks.

The rest of this work is organized as follows: Section 2 briefly describes existing network structures related to the proposed structure; Section 3 describes the proposed method in detail; Section 4 provides the results and analysis of different experiments; finally, Section 5 presents our conclusions and recommendations for future work.

## 2. Related Work

### 2.1. CNN-Based Super-Resolution of Single Images

SRCNN is the first CNN-based super-resolution method with a simple network model; however, its use of interpolation operations may affect the performance of the network. FSRCNN [20] uses a deconvolution layer on top of SRCNN to enlarge the image and improve its resolution. VDSR [21] improves performance by stacking deeper convolutional layers, although the resulting increase in the number of network layers leads to greater computational complexity. ESPCN [22] contains a sub-pixel convolutional layer at the end of the network to handle the image super-resolution reconstruction problem with different scale factors, and additionally offers faster processing speeds.

LapSRN [23] improves reconstruction quality through the residual pyramid frame, although it requires transformation of the pyramid frame into multiple parts for processing, which increases computational complexity. In contrast, CMSC [24] can improve both computational efficiency and reconstruction quality; however, it is not effective for image edge detail reconstruction. To further improve the reconstruction accuracy of edge details, MSRN [25] uses a multi-scale residual structure and a densely connected layer to reconstruct the edge details of images more accurately. However, because MSRN requires more computational complexity, a trade-off between computational efficiency and reconstruction accuracy is needed when selecting a suitable model in practical applications.

Among the common recurrent networks, DRCN [26] and DRRN [27] are suitable for input images of different sizes, the former enhancing the feature representation by recursive connectivity and upsampling and the latter avoiding loss of feature information by introducing residual and cyclic structures. DSRN [19] and DRUDN [28] can both process input images of different sizes, with the former predicting high-resolution images through Laplace pyramids and the latter improving the sharpness and quality of images through recursive upsampling and denoising techniques. However, recursive connections in DRCN leads to an increase in computation, and the residual structure in DRRN can only handle local features. Additionally, the Laplace pyramid in DSRN requires a large amount of computation and storage space, while the denoising technique in DRUDN may eliminate some useful detailed information.

### 2.2. Multi-Scale Networks

**Figure 1:**
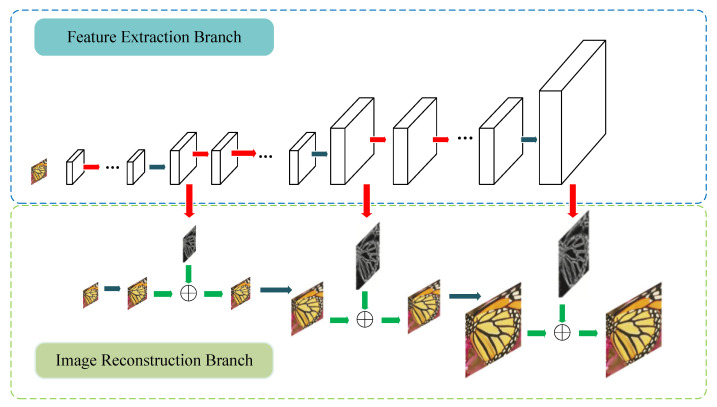
Schematic diagram of LapSRN network; red arrow: convolution for feature extraction, blue arrow: inverse convolution for resolution enhancement, green arrow: pixel-by-pixel summation.

Multi-scale networks can achieve multi-scale feature representation of images by processing blocks of images at different scales, which improves the accuracy and efficiency of the network [30]. In super-resolution tasks, the loss of detail information in low-resolution images is severe, limiting the quality of the reconstructed images [31]. To capture the features of low-resolution images more comprehensively and accurately, a multi-scale network can be used to extract features at different scales and reconstruct images with rich details and clear textures. Figure 1 shows the simplified structure of the LapSRN multi-scale network module.

LapSRN incorporates a Laplace pyramid into the CNN structure, with low-resolution images being input directly into the network and clear HR images reconstructed via gradual upsampling. The structure of the LapSRN can be thought of as having multiple stages, with each stage accomplishing a two-fold upsampling operation in which features are first extracted through a number of convolutional layers and then the sizes of the extracted features undergo twofold upsampling through the deconvolutional layers. In each stage, the features are first extracted using a number of convolutional layers. In LapSRN, subnetworks of different scales share parameters, allowing the number of parameters of the model to be greatly reduced and the training speed of the model to be improved. However, the model is relatively complex, and this structure can only accomplish the task of super-resolution reconstruction to the power of two. Despite the shortcomings of LapSRN, the multiscale idea allows the model to process information at different scales simultaneously, which helps to improve performance on image processing tasks.

### 2.3. Recursive-Based SISR Method

**Figure 2:**
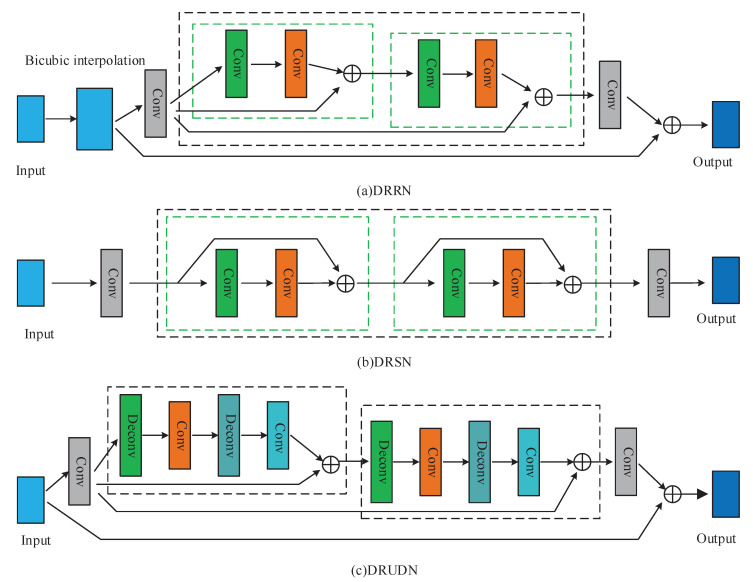
The structures of recursive networks: simplified network models for (**a**–**c**) DRRN, DSRN, and DRUDN, respectively.

Recursive networks are widely used in SISR in order to reduce the number of parameters in SISR methods. This section focuses on the three recurrent networks most relevant to the proposed method: DRRN [27], DSRN [19], and DRUDN [28]. Figure 2 shows the simplified structures of these recurrent networks.

DRRN: In Figure 2a, the green dashed boxes indicate the number of residual blocks in the recursive network, while the black boxes indicate the number of recursive networks. The network learns through the design idea of deep residual networks by stacking multiple residual blocks together to form a deep network structure. It incorporates both local and global residual learning to construct a deep network, thereby reducing the burden of image information in the deep network structure and making the network easier to train. In addition, recursive learning is introduced in the recursive block to keep the model compact and reduce the number of parameters. DRRN uses local normalization and global normalization to enhance the generalization ability of the network.

DSRN: In Figure 2b, the green dashed boxes indicate the number of residual units and the black boxes indicate the number of recursive networks. DSRN is different from many other networks in that the LR images are input directly into the network instead of interpolating images, thereby reducing its computational complexity. Another difference between DSRN and DRRN is that DSRN uses the output of the previous recursive unit as the local residual instead of the global input, ignoring the hierarchical feature information.

DRUDN: In Figure 2c, the black boxes in the figure represent recursive upsampling and downsampling blocks. DRUDN employs a strategy of feeding low-resolution (LR) images directly into the network and extends the sensory field of the entire network using convolutional and deconvolutional layers, which effectively improves the performance of super-resolution reconstruction. The core of this method lies in the fact that by introducing convolution and deconvolution operations, the network is able to capture image features more comprehensively, which in turn enhances its ability to learn details. Th use of an attention mechanism to process the important information in the image has been proposed.

### 2.4. Attention Mechanism

Attention mechanisms have attracted the attention of many scholars because they allow models to focus more flexibly on certain features or regions when processing input data, improving the performance and capability of the resulting models [32,33,34,35]. First, Hu et al. [32] proposed the SE attention mechanism to achieve significant performance gains in a variety of tasks, greatly facilitating the application of attention mechanism in computer vision tasks. RCAN integrated channel attention (CA) into the residual block, which pays more attention to the high-frequency channel features to improve the reconstruction performance. Wang et al. [33] proposed a combination of spatial attention and channel attention in the Convolutional Block Attention Module (CBAM), which captures more informative features at all locations by learning channel and spatial correlation features at each layer. The Holistic Attention Network (HAN) [34] introduced a Layer Attention Module (LAM) to learn the correlation between features in multi-scale layers. Attention mechanisms in CNNs are able to redistribute the network’s attention to different parts or features in order to enhance the processing of the information that is most of interest while improving network performance.

**Figure 3:**
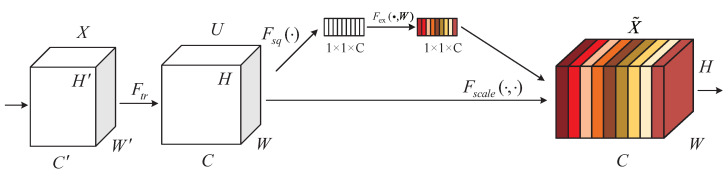
SEnet network structure.

Squeeze-and-Excitation Networks (SEnet), which aim to focus on the relationship between different channels, automatically learn the importance of different channel features. They selectively enhance those features containing useful information and suppress useless features based on the global information. The basic structure of SEnet is shown in Figure 3, in which a feature map *X* with channel number $C^{{}^{\prime }}$, height $H^{{}^{\prime }}$, and width $W^{{}^{\prime }}$ is input and a feature map *U* with channel number *C*, height *H*, and width *W* is obtained by one or more standard convolution operations.

The steps of SEnet are as follows. First, the squeeze operation uses global average pooling to encode the entire spatial feature on a channel as a global feature, i.e., the input of H×W×C is converted to an output of 1×1×C. The excitation operation is a nonlinear transformation of the result after the squeeze operation using two fully connected neural networks to obtain the magnitude of the importance of different channels. Finally, the scale operation, i.e., the weighting operation, weights the output weights after the excitation operation to the previous features by multiplying them channel-by-channel to complete the rescaling of the original features in the channel dimension and obtain the feature map $\tilde{X}$ with dimensions H×W×C. Using the SEnet model, the overall channel features are enhanced and the network performance is improved.

## 3. Proposed Method

### 3.1. Overview of the Network Model

**Figure 4:**
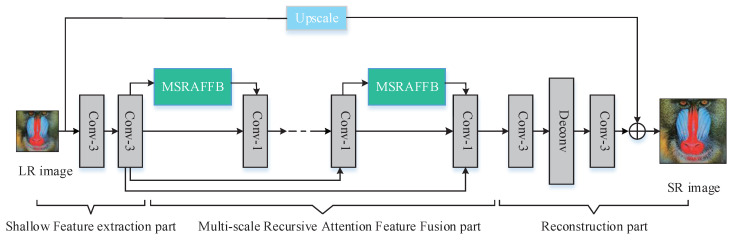
Network structure of the multi-scale recursive attention feature fusion network.

MSRAFFN contains three parts: a shallow feature extraction module, a multi-scale recursive attention feature fusion module, and a reconstruction module. In this structure, the shallow feature extraction unit extracts global features through two convolutional layers, which lays the foundation for subsequent deep feature extraction. MSRAFFB is used to extract the deep features, which contains multiple branches; each branch outputs features with different scales and applies SEnet to learn deep feature information and then fuse the feature information of different scales for improved network performance. The residual connection method in the backbone network can integrate shallow features and deep features via cross-layer connection, allowing the layered features from the multi-scale feature extraction module to be effectively used to extract richer and more accurate feature information from low-resolution images while retaining more detailed information for better reconstruction of high-resolution images. Finally, the reconstruction module generates residual images using general convolution and deconvolution operations to further improve the quality of image reconstruction. The structure of MSRAFFN is shown in Figure 4.

Table 1 shows the detailed architecture of the network, illustrating the number of convolutional layers used in the CNN network. The input low-resolution image is first processed by two 3 × 3 convolutions, then we several MSRAFFBs with the same structure are used to form the backbone of the network and fuse the processed features of the front-end network using a 1 × 1 convolution after each MSRAFFB. The MSFE contains several branches internally, with the first one containing only one 3 × 3 convolutional layer and the others containing one 3 × 3 convolutional layer and a 1 × 1 convolutional layer each. The AFF unit includes two ordinary convolutional layers, one pooling layer, and two fully connection layers. The reconstruction module includes two ordinary convolutional layers and one deconvolution layer.

### 3.2. Shallow Feature Extraction Module

In order to speed up the training of super-resolution models, mainstream shallow feature extraction methods adopt the strategy of directly inputting small-sized low-resolution images into the shallow feature extraction module instead of first amplifying the images by interpolation before inputting them into the network. This strategy reduces the computational complexity of the network and accelerates the training speed. Inspired by this idea, our method uses a similar approach to extract shallow image features; specifically, the shallow feature extraction module in the model uses two 3 × 3 convolutional layers (the padding and stride are both 1) to extract the shallow features of the image. This simple feature extraction method can effectively reduce the training time of the super-resolution model, retain all the details and complexity in the original image as much as possible, and make the generated high-resolution image more similar to the ground truth image. The input LR image is denoted as $I_{LR}$, while the extracted shallow features are denoted as $F_{0}$:

(1) $$F_{0}=H_{FEP}\left(I_{LR}\right)$$

where $H_{FEP}$(•) denotes the shallow feature extraction part.

### 3.3. Multi-Scale Recursive Attention Feature Fusion Module

**Figure 5:**
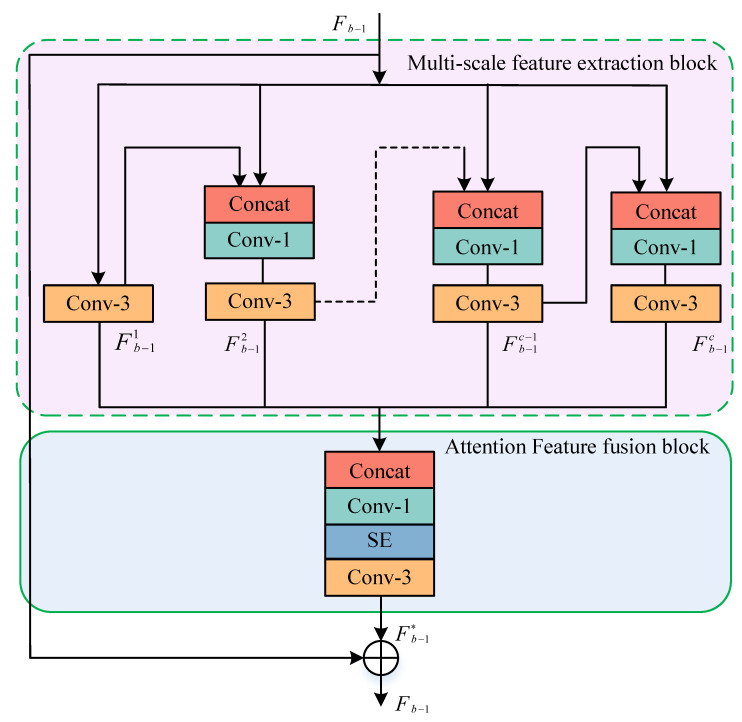
Structure of the multi-scale attention feature fusion module; the pink shaded portion is the MSFE block and the blue shaded portion is the AFF block.

The MSRAFFB is an important module of the designed algorithm to extract deep feature information. As shown in Figure 5, MSRAFFB is composed of two parts, MSFE and AFF. The AFF unit takes the output of all branches as input, adaptively fuses multi-scale features, and learns deep feature information through SEnet to improve network performance. In the backbone network, the shallow features are integrated with the deep features by means of cross-layer connections using residual connections and the hierarchical features of MSRAFFB are used effectively in a layer-wise manner, meaning that we have

(2) $$F_{DF}=H_{MRA}\left(F_{0}\right).$$

where $F_{DF}$ denotes the deep feature information and $H_{MRA}$(•) denotes the multi-scale recursive attention feature fusion part.

The multi-scale feature extraction unit mainly consists of 3 × 3 (where the padding and stride are both 1) and 1 × 1 (padding 0, stride 1) convolutional layers, and contains numerous parallel branches inside which connect the input of MSRAFFB and the output of the previous branch as the input of the next branch through the concatenation operator. The output of each branch has a different perceptual field, as follows:

(3) $$\begin{array}{c} F_{b-1}^{1}=f_{3\;\times \;3}\left(F_{b-1}\right) \end{array}$$

(4) $$\begin{array}{c} F_{b-1}^{2}=f_{3\;\times \;3}\left(f_{1\;\times \;1}\left(concat\left(F_{b-1},F_{b-1}^{c}\right)\right)\right) \end{array}$$

⋮

(5) $$\begin{array}{c} F_{b-1}^{c-1}=f_{3\times 3}\left(f_{1\times 1}\left(concat\left(F_{b-1},F_{b-1}^{c-2}\right)\right)\right) \end{array}$$

(6) $$\begin{array}{c} F_{b-1}^{c}=f_{3\times 3}\left(f_{1\times 1}\left(concat\left(F_{b-1},F_{b-1}^{c-1}\right)\right)\right). \end{array}$$

where $F_{b-1}^{1}$ represents the output of the bth MSRFFB block. $f_{3\times 3}$ is the 3 × 3 convolution, $f_{1\;\times \;1}$ is the 1 × 1 convolution, concat(•) is the concatenation operator, and $F_{b-1}^{c}$ represents the output of the *c*-th branch of the *b*-th MSRFFB block. The attention feature fusion unit connects the different scale features in each branch by the connection operator, then enhances the overall channel features using SEnet to improve the network performance; thus, the output of this unit is

(7) $$F_{b}^{*}=f_{3\;\times \;3}\left(f_{SE}\left(f_{1\;\times \;1}\left(concat\left(F_{b-1}^{1},F_{b-1}^{2},\cdots ,F_{b-1}^{c}\right)\right)\right)\right).$$

In addition, a residual connection is used to connect the initial input features to the output of the attention feature fusion unit, making the final output of this module

(8) $$F_{b}=F_{b}^{*}+F_{b-1}.$$

### 3.4. Reconstruction Module

**Figure 6:**
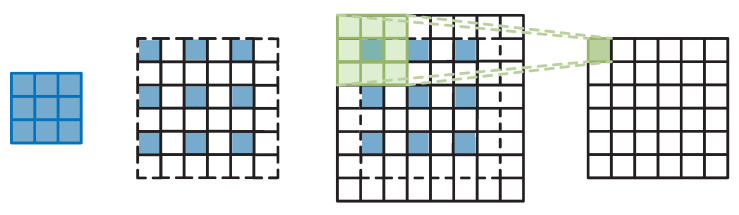
Deconvolution model at ×2 SR.

The reconstruction module is shown in Figure 4; it contains two 3×3 convolutions and one deconvolution. The final reconstructed image is obtained by using the residual module to concatenate the image after double triple interpolation and fuse it with the convolution output. The first ordinary convolutional layer with 64 convolutional kernels is used for mapping the feature map to a super-resolution image. Deconvolution is used to upsample the image by scaling the low-resolution image to a high-resolution level. The last ordinary convolutional layer is used to recover the number of channels in order to generate a three-channel RGB image. The deconvolution layer parameters are set as follows: upscale factors of ×2, stride of 2, padding of 2, and convolution kernel size of 6. For upscale factors of ×3, the stride is 3, the padding is 2, and the convolution kernel size is 7. For upscale factors of ×4, the stride is 4, the padding is 2, and the convolution kernel size is 8. When the scaling factor is 2, the deconvolution model is as shown in Figure 6, which first expands the input image to twice the original size and sets all the new pixel values to 0. Then, the deconvolution operation is performed using the convolution (padding 0, stride 1) to upsample the low-resolution image in order to obtain a higher-resolution feature map.

### 3.5. Loss Function

The $L_{2}$ loss function may over-penalize the squared value of the error, resulting in the loss of high-frequency detail information in the image. On the other hand, the $L_{1}$ loss function can effectively penalize the absolute error between the predicted and true values, causing the network to be more concerned with the recovery of details in the image. Therefore, the $L_{1}$ loss function is chosen for use in the design of the super-resolution network. If there are *N* training samples, the $L_{1}$ loss function can be expressed as follows:

(9) $$L_{Loss}=\frac{1}{N}\sum_{i=1}^{N}∥F\left(X_{i}\right)-Y_{i}∥$$

where $Y_{i}$ denotes the original high-resolution image, $F(x_{i})$ denotes the reconstructed super-resolution image, $∥F\left(x_{i}\right)-Y_{i}{∥}_{1}$ denotes the loss of the *i*-th original image and the reconstructed image, and the loss is averaged over all images.

## 4. Experimental Results and Analysis

The experimental system was an Ubuntu 20.04 server with an NVIDIA GP102 GPU; the model was implemented using the PyCharm compilation platform in Python language. In order to objectively evaluate the performance of the proposed method, we used two metrics that are recognized as authoritative in the field of image processing, namely, the peak signal-to-noise ratio (PSNR) [36] and the structural similarity (SSIM) [37]. These objective evaluation metrics allow for a more objective performance assessment of the proposed method.

### 4.1. Experiment Details

The DIV2K [38] dataset, which contains 800 high-quality images, was used for training in the experiments. During the training phase, randomly cropped 48 × 48 LR image patches were used as input to the network model and the patch size of HR images was determined based on the scale factor. The patches were randomly added using horizontal, vertical, flipped, and 90° rotated data enhancement techniques to expand the diversity and number of samples in the dataset. In addition, the Adam optimizer [39] was used with an initial learning rate set to 0.0002 and a 50% reduction every 200 epoch. In the network structure, the number of output channels of the last convolutional layer was set to 3 in order to adapt to the reconstruction of RGB images. The number of output channels for the rest of the 3 × 3 convolutional layers was set to 64, with the PReLU activation function, padding of 0, and stride of 1. For the proposed multi-scale network model, we used five benchmark datasets in the performance evaluation: Set5 [40], Set14 [41], B100 [42], Urban100 [43], and Manga109 [44]. The Set5, Set14, and BSD100 datasets consist of natural scenes, the Urban100 dataset contains urban scenes such as roads and buildings, and the Manga109 dataset contains many manga pictures. These datasets are widely used in the field of image super-resolution, and allow for comprehensive performance testing of the proposed algorithms in different scenarios.

### 4.2. Network Model Parameters Analysis

**Figure 7:**
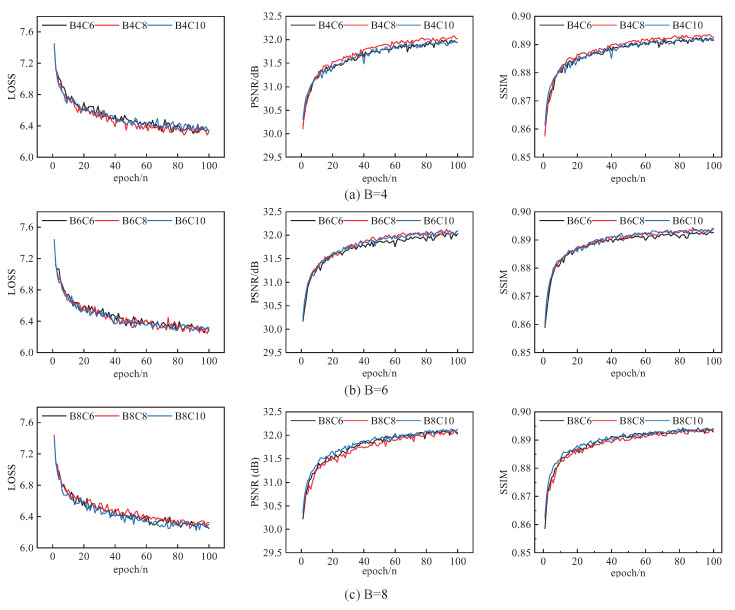
Effect of different values of C on network performance: (**a**) network convergence analysis for B = 4; (**b**) network convergence analysis for B = 6; and (**c**) network convergence analysis for B = 8.

In this section, we explore the effect on network performance of the number of MSRAFFB modules *B* in MSRAFFN and the number of branches *C* in MSFE. Experiments were performed on the DIV2K dataset, setting the scaling factor to ×4 and the number of iterations to 100. the values of *B* were 4, 6, and 8, while the values of *C* were 6, 8, and 10. To test the effect of the *C* parameter, the value of *B* was fixed and the value of *C* was set to 6, 8, and 10. The experimental results are shown in Figure 7.

From Table 2, it can be seen that when B = 4, 6, and 8, the PSNR on all five datasets increases and then decreases with the increase of C. This indicates that increasing the number of branches in the module does not always ensure that the performance of the network is improved; based on this comparison, C should first be set to 8. In order to explore the effect of the value of B on network performance, C was set to 8 and B was set to 4, 6, and 8. The performance of the model did not always improve with higher values; it can be seen that when the value of B is increased from 8 to 10, both the PSNR and SSIM metrics decrease. These experimental results show that stacking a greater number of modules does not always lead to improved network performance. Thus, the value of B was set to 6.

From Figure 7a, it can be seen that when B = 4, the lowest LOSS value is obtained when C = 8, and that the largest PSNR and SSIM values are obtained in this case. In Figure 7, when B = 6, the PSNR value is the lowest when C is taken as 6. When C is taken as 8 or 10, the difference between the two in terms of performance is not obvious. The network performance when B = 8 is shown in Figure 7c. It can be seen that the LOSS value is the lowest at C = 10. When the number of iterations increases to a certain level, the difference between the PSNR and SSIM values at C = 6 and C = 10 becomes smaller.

While the network performance of B4C8, B6C8, B6C10, B8C6, and B8C10 may be better when the value of B is fixed, considering the overall network complexity, B4C8, B6C8, and B8C6 were finally selected.

**Figure 8:**
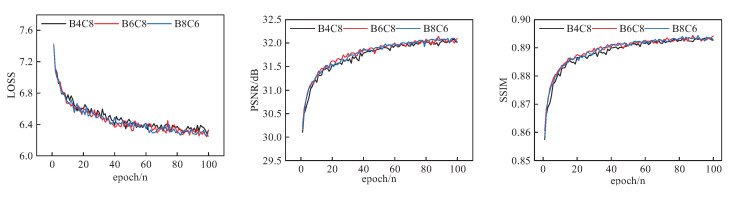
Performance comparison of B4C8, B6C8, and B8C6.

As shown in Figure 8, by comparing the corresponding LOSS values of B4C8, B6C8 and B8C6, it is apparent that B6C8 and B8C6 have the lowest LOSS values and that their PSNR and SSIM values are higher. Compared to B4C8, B6C8 and B8C6 both have better network performance. Based on the analysis of Table 2 and Figure 7 and Figure 8, which show the trade-off relationship between network performance and model size, B = 6 and C = 8 were finally selected.

### 4.3. Comparison with State-of-the-Art Methods

To enhance the comprehensiveness of this study, Table 3 shows the main differences between our proposed algorithm and typical SR algorithms in terms of upsampling methods, including different recursive networks, residual networks, and loss functions. The upsampling methods are categorized into three types: bicubic interpolation, deconvolution, and subpixel convolution, while the loss functions can be categorized into $L_{1}$, $L_{2}$, MSE, and char loss functions. Our proposed algorithm uses deconvolution to enlarge the target image, utilizes the ideas of recursive and residual networks to improve the network’s ability to extract feature information, and uses the $L_{1}$ loss function to optimize the network model.

To test the performance advantages and disadvantages of the MSRAFFA model, the super-resolution reconstruction model proposed in this paper was compared with existing super-resolution reconstruction models on five benchmark test sets. The tested reconstruction models were SRCNN, FSRCNN, VDSR, DRCN, LapSRN, DRRN, DSRN, IDN, and MSRN.

PSNR and SSIM are authoritative evaluation metrics that can be used to objectively assess image quality. These two metrics were compared at scale factors of ×2, ×3, and ×4. For the IDN method, this experiment was conducted using only the metrics in its original text for comparison, and the network model was not evaluated on the Manga109 dataset. The results are shown in Table 4, where the best performance is indicated in bold and the second-best by underlining. It can be seen in the table that with ×2 SR, the performance of our proposed method is not much different from that of MSRN compared to the previous methods. With ×3 SR, the PSNR values of our proposed method are improved by 0.19 dB and 0.37 dB compared to those of MSRN on the Urban100 and Manga109 datasets, respectively. With ×4 SR, the PSNR values of our proposed method are improved by 0.19 dB and 0.79 dB compared to those of MSRN on the Urban100 and Manga109 test sets, respectively. Finally, the PSNR values of our proposed method are improved by 0.19 dB and 0.79 dB on the Urban100 and Manga109 datasets, respectively, compared to the PSNR values of MSRN. The results of these experiments prove the superior performance of our proposed method.

**Figure 9:**
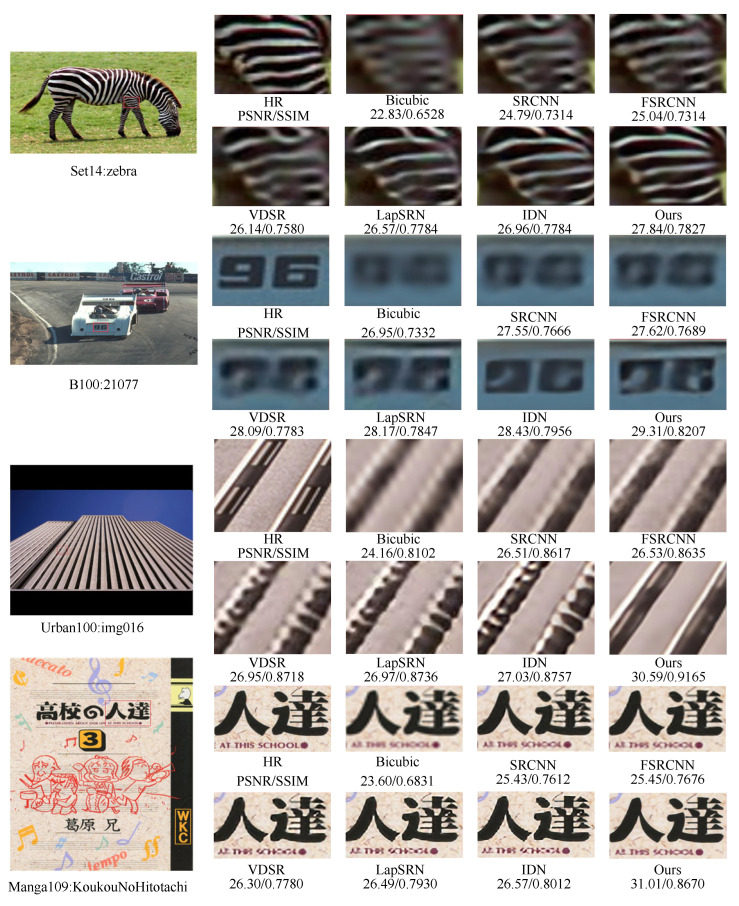
Comparison of the proposed method with other methods in terms of subjective visualization on the Set14, B100, Urban100, and Manga109 datasets at ×4 SR.

In order to show the difference in subjective visual effects between the proposed method and existing methods, we chose a scaling factor of 4 and selected a representative image in the Set14, BSD100, Manga109, and UrBan100 datasets for a local magnification comparison. The visual comparison of image effects is shown in Figure 9. A comparison of the four reconstructed images with local enlarged parts shows that the reconstructed images from Bicubic, SRCNN, and FSRCNN are blurred and do not reconstruct the clear detailed texture contours of the images. The VDSR, LapSRN, and IDN methods are more or less able to reconstruct the texture contours of images. Compared with the above methods, our proposed model can reconstruct more texture contour information, and the reconstructed images are clearer and sharper with better visual perception.

As can be seen from the example of Set14: zebra, our proposed model is able to reproduce the textured parts of the animal’s body more clearly. From the example of Urban100: img016, although our proposed model does not completely reconstruct all the details of the building outline, the outline of the reconstructed building is clearer and more textured.

### 4.4. Ablation Study

To test the effectiveness of MSFE and AFF in improving the performance of the model, we conducted an ablation experiment. Performance was examined using a common convolution instead of the corresponding module. To eliminate any performance gain caused by different parameters, it was ensured that all networks contained the same number of MSRAFFBs and used same training strategy. The models for the different ablation studies were as follows:

(1) Normal convolution replacing the corresponding MSFE module, denoted as Non MSFE.
(2) A 3 × 3 standard convolution replacing the corresponding AFF module, denoted as Non-AFF.
(3) Both the MSFE and AFF modules are present, denoted as MSFE + AFF.

**Figure 10:**
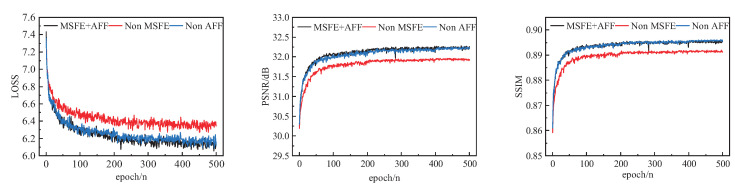
The performance comparison of Non-MSFE, Non-AFF, and MSFE + AFF on the Set5 (×4) dataset.

The training results after 500 iterations are shown in Table 5 and Figure 10.

It can be visually observed in Figure 10 that the model performance degrades very significantly without the multi-scale feature extraction module, with lower PSNR and SSIM values and larger LOSS values. The attention feature extraction module has a similar impact. As can be seen from Table 5, the network with the multi-scale feature extraction module obtains better results compared to the reconstruction with the ordinary convolution kernel. The average PSNR values on the test set are improved by 0.31 dB, 0.26 dB, 0.18 dB, 0.51 dB, and 1.06 dB on Set5, Set14, B100, Urban100m and Manga109, respectively. With the AFF module, the average PSNR values on the Set5, Set14, B100, Urban100, and Manga109 test sets improve by 0.03 dB, 0.03 dB, 0.02 dB, 0.07 dB, and 0.41 dB, respectively, compared with normal convolution.

**Figure 11:**
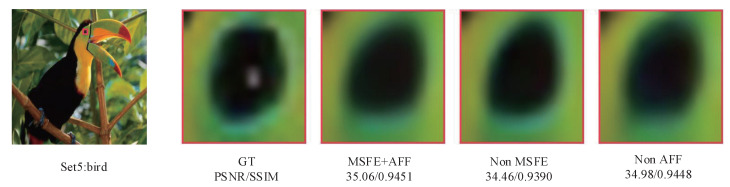
Visual comparison of the Non-MSFE, Non-AFF, and MSFE + AFF on the Set5 (×4) dataset.

In Figure 11, it can be seen that the eyes of the bird reconstructed by MSFE+AFF are clearer and have more detail. The results of the ablation experiments show that deeper feature information can be extracted using MSFE and AFF, and that this rich feature information can be fully utilized to improve the quality of the reconstructed images.

### 4.5. Model Performance Analysis

**Figure 12:**
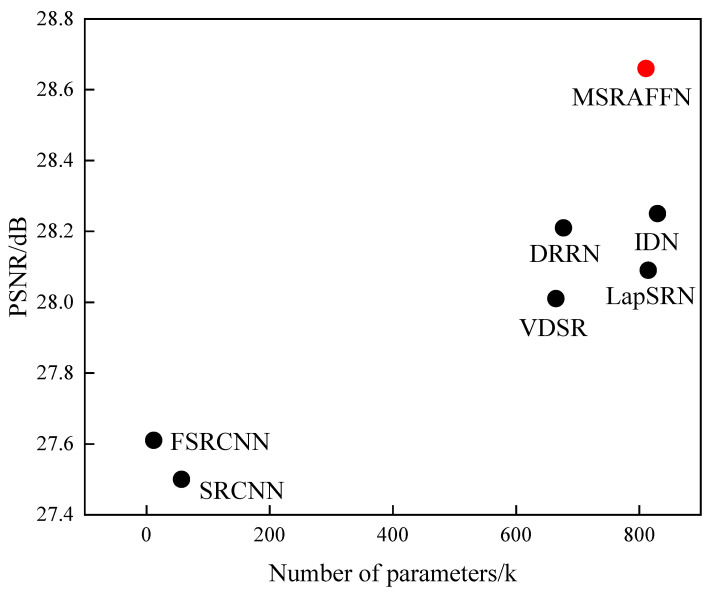
Model performance compared to the number of parameters on the Set14 dataset with a ×4 scaling factor.

While our method shows promising results regarding image quality metrics, it is essential to consider its computational efficiency as well. To further demonstrate the excellence of the MSRAFFN algorithm, we compared the number of parameters of each network using the PSNR evaluation metrics; the selected dataset was the Set14 dataset with a scaling factor of 4. The results are shown in Figure 12. It is evident from the figure that the MSRAFFN model has a larger number of parameters compared to algorithms such as DRRN and VDSR; however, it achieves significantly higher reconstruction performance. It is worth noting that even though IDN has a larger number of parameters than our proposed MSRAFFN algorithm, its reconstruction performance is much lower.

## 5. Conclusions and Future Prospects

In this paper, we have proposed MSRAFFN for super-resolution image reconstruction. The advantage of this model is that MSRAFFB is embedded in the network to achieve the extraction of features at different scales, then fully fuse the features at different scales to recover the rich details of the image. A channel attention mechanism is introduced on the basis of feature fusion to enhance the channel features of the overall network. In addition, residual connectivity is used to integrate shallow and deep features via cross-layer connectivity, which reduces the loss of shallow feature information in the process of propagation to deeper levels while fully utilizing the features at each intermediate level. Finally, the low-resolution image is restored to the high-resolution level using deconvolution.

Through extensive experiments and analyses on five benchmark datasets, we have shown that our proposed model has certain advantages over current advanced models in terms of objective indicators. It shows better reconstruction effects in terms of subjective vision, and most of the reconstructed images have clearer and more realistic texture contours. In the future, we intend to devote our research efforts to seeking a more general and effective image reconstruction model. Notably, the proposed model is mainly intended for use in processing natural images; extending it to other scenes, such as medical images, satellite images, etc., is a problem that needs to be explored in future research.

## Figures and Tables

**Table 1:** Detailed network architecture.

Layer	Kernel Size
Shallow feature extraction module	3 × 3 × 64, 3 × 3 × 64
Multi-scale feature extraction unit	3 × 3 × 64, 3 × 3 × 64, 1 × 1 × 128
	3 × 3 × 64, 1 × 1 × 128, 3 × 3 × 64, 1 × 1 × 128
	3 × 3 × 64, 1 × 1 × 128, 3 × 3 × 64, 1 × 1 × 128
	3 × 3 × 64, 1 × 1 × 128, 3 × 3 × 64, 1 × 1 × 128
Attention feature fusion unit	1 × 1 × 512
	Average_pool, fc1, fc2
	3 × 3 × 64
	1 × 1 × 128
Reconstruction module	3 × 3 × 64
	Deconvolution
	3 × 3 × 64 × 3

**Table 2:** Mean PSNR/SSIM on the five datasets(×4 SR) when B is taken 4, 6, and 8 and C is taken 6, 8, and 10. The best and second-best results are indicated by bold and underline, respectively.

Different Methods	Set5	Set14	B100	Urban100	Manga109
	**PSNR/SSIM**	**PSNR/SSIM**	**PSNR/SSIM**	**PSNR/SSIM**	**PSNR/SSIM**
B4C6	31.99/0.8924	28.43/0.7777	27.45/0.7322	25.74/0.7747	30.03/0.9029
B4C8	32.08/0.8936	28.48/0.7790	27.48/0.7331	25.83/0.7779	30.12/0.9045
B4C10	31.36/0.8890	28.26/0.7747	27.33/0.7284	25.57/0.7693	29.41/0.8962
B6C6	32.05/0.8926	28.49/0.7781	27.49/0.7322	25.82/0.7758	30.08/0.9036
B6C8	**32.28/0.8954**	**28.66/0.7828**	**27.61/0.7368**	**26.23/0.7895**	**30.96/0.9108**
B6C10	32.12/0.8939	28.48/0.7795	27.48/0.7338	25.84/0.7785	30.18/0.9050
B8C6	32.10/0.8943	28.53/0.7795	27.50/0.7332	25.86/0.7782	30.24/0.9061
B8C8	32.09/0.8939	28.47/0.7791	27.50/0.7333	25.85/0.7780	30.13/0.9045
B8C10	31.74/0.8894	28.34/0.7768	27.37/0.7298	25.54/0.7762	30.18/0.9050

**Table 3:** Comparison with classical methods in terms of upsampling method, use of recurrent and residual networks, and loss function. The √ means that a method was used, while a blank space means that it was not used.

Methods	Upsampling Methods	Recursive Network	Residual Network	Loss Function
SRCNN	bicubic			$L_{2}$
FSRCNN	deconvolution		√	$L_{2}$
VDSR	bicubic		√	MSE
DRCN	bicubic	√	√	$L_{2}$
LapSRN	deconvolution		√	Char
IDN	deconvolution		√	MSE
MSRN	subpixel convolution		√	$L_{1}$
Ours	deconvolution	√	√	$L_{1}$

**Table 4:** Quantitative evaluation (PSNR(dB)/SSIM) of different SR algorithms with ×2, ×3 and ×4; the best results are indicated by bold and the second-best results by underline.

Method	Scale	Set5	Set14	B100	Urban100	Manga109
		**PSNR/SSIM**	**PSNR/SSIM**	**PSNR/SSIM**	**PSNR/SSIM**	**PSNR/SSIM**
Bicubic		33.66/0.9299	30.24/0.8668	29.56/0.8431	26.88/0.8403	30.80/0.9339
SRCNN [16]		36.66/0.9542	32.45/0.9067	31.36/0.8879	29.50/0.8946	35.60/0.9663
FSRCNN [20]		37.00/0.9558	32.63/0.9088	31.53/0.8920	29.88/0.9020	36.67/0.9710
VDSR [21]		37.53/0.9587	33.03/0.9124	31.90/0.8960	30.76/0.9140	37.22/0.9750
DRCN [26]		37.63/0.9588	33.04/0.9118	31.85/0.8942	30.75/0.9133	37.55/0.9732
LapSRN [23]	× 2	37.52/0.9591	32.99/0.9124	31.80/0.8949	30.41/0.9101	37.53/0.9740
DRRN [27]		37.74/0.9591	33.23/0.9136	32.05/0.8973	31.23/0.9188	37.88/0.9749
DSRN [19]		37.66/0.9590	33.15/0.9130	32.10/0.8970	30.97/0.9160	-/-
IDN [18]		37.83/0.9600	33.30/0.9148	32.08/0.8985	31.27/0.9196	-/-
MSRN [25]		**38.08/0.9605**	**33.74/0.9170**	**32.23/0.9013**	**32.22/0.9326**	38.82/**0.9868**
Proposed Method		38.03/**0.9607**	33.58/**0.9178**	32.18/0.8998	**32.22/0.9289**	**38.83/0.9776**
Bicubic		30.39/0.8682	27.55/0.7742	27.21/0.7385	24.46/0.7349	26.95/0.8556
SRCNN [16]		32.75/0.9090	29.30/0.8215	28.41/0.7863	26.24/0.7989	30.48/0.9117
FSRCNN [20]		33.18/0.9140	29.37/0.8240	28.53/0.7910	26.43/0.8080	31.10/0.9210
VDSR [21]		33.66/0.9213	29.77/0.8314	28.82/0.7976	27.14/0.8279	32.01/0.9340
DRCN [26]		33.82/0.9226	29.76/0.8311	28.80/0.7963	27.15/0.8276	32.24/0.9343
LapSRN [23]	× 3	33.82/0.9227	29.79/0.8320	28.82/0.7973	27.07/0.8271	32.21/0.9350
DRRN [27]		34.03/0.9244	29.96/0.8349	28.95/0.8004	27.53/0.8375	32.71/0.9379
DSRN [19]		33.88/0.9220	30.26/0.8370	28.81/0.7970	27.16/0.8280	-/-
IDN [18]		34.11/0.9253	29.99/0.8354	28.95/0.8013	27.42/0.8359	-/-
MSRN [25]		34.38/0.9262	30.34/0.8395	29.08/0.8041	28.08/**0.8554**	33.44/0.9427
Proposed Method		**34.48/0.9282**	**30.35/0.8421**	**29.11/0.8059**	**28.27/0.8543**	**33.81/0.9460**
Bicubic		28.42/0.8104	26.00/0.7027	25.96/0.6675	23.14/0.6577	24.89/0.7866
SRCNN [16]		30.48/0.8628	27.50/0.7513	26.90/0.7101	24.52/0.7221	27.58/0.8555
FSRCNN [20]		30.72/0.8660	27.61/0.7550	26.98/0.7150	24.62/0.7280	27.90/0.8610
VDSR [21]		31.35/0.8838	28.01/0.7674	27.29/0.7251	25.18/0.7524	28.83/0.8870
DRCN [26]		31.53/0.8854	28.02/0.7670	27.23/0.7233	25.14/0.7510	28.93/0.8854
LapSRN [23]	× 4	31.54/0.8866	28.09/0.7694	27.32/0.7264	25.21/0.7553	29.09/0.8900
DRRN [27]		31.68/0.8888	28.21/0.7721	27.38/0.7284	25.44/0.7638	29.45/0.8946
DSRN [19]		31.40/0.8830	28.07/0.7700	27.25/0.7240	25.08/0.7470	-/-
IDN [18]		31.82/0.8903	28.25/0.7730	27.41/0.7297	25.41/0.7632	-/-
MSRN [25]		32.07/0.8903	28.60/0.7751	27.52/0.7273	26.04/**0.7896**	30.17/0.9034
Proposed Method		**32.28/0.8954**	**28.66/0.7828**	**27.61/0.7368**	**26.23/0.7895**	**30.96/0.9108**

**Table 5:** Average PSNR and SSIM of the three models on the five datasets (×4).

Method	Set5	Set14	B100	Urban100	Mnaga109
	**PSNR/SSIM**	**PSNR/SSIM**	**PSNR/SSIM**	**PSNR/SSIM**	**PSNR/SSIM**
MSFE + AFF	32.28/0.8954	28.66/0.7828	27.61/0.7368	26.23/0.7895	30.96/0.9108
Non MSFE	31.97/0.8918	28.40/0.7772	27.43/0.7312	25.72/0.7732	29.90/0.9015
Non AFF	32.25/0.8960	28.63/0.7827	27.59/0.7369	26.16/0.7886	30.55/0.9098

## Data Availability

The dataset is available upon request from the authors via email at hxw_1405@163.com.
